# Systematic review: Clinical outcomes of discontinuation of oral antivirals in hepatitis B-related liver cirrhosis

**DOI:** 10.3389/fpubh.2022.1037527

**Published:** 2022-11-03

**Authors:** Yuhao Yao, Jiaxin Zhang, Xiaoke Li, Xiaobin Zao, Xu Cao, Guang Chen, Yong'an Ye

**Affiliations:** ^1^Department of Gastroenterology, Dongzhimen Hospital, Beijing University of Chinese Medicine, Beijing, China; ^2^First School of Clinical Medicine, Beijing University of Chinese Medicine, Beijing, China; ^3^Institute of Liver Diseases, Beijing University of Chinese Medicine, Beijing, China; ^4^Key Laboratory of Chinese Internal Medicine of Ministry of Education and Beijing, Dongzhimen Hospital, Beijing University of Chinese Medicine, Beijing, China

**Keywords:** chronic hepatitis B, discontinuation, nucleos(t)ide analog, cirrhosis, review—systematic

## Abstract

**Background:**

Discontinuation of Nucleos(t)ide analogs (NAs) remains one of the most controversial topics in the management of hepatitis B-related liver cirrhosis. However, clinical outcomes after NAs discontinuation have not been studied.

**Aim:**

The aim of this systematic review is to evaluate existing data on clinical outcomes of NAs withdrawal in chronic hepatitis B (CHB) patients with cirrhosis.

**Methods:**

A literature search (until May 2022) was performed in order to identify all published studies including hepatitis B-related cirrhotic patients who discontinued NAs in virological remission with off-therapy follow-up >12 months.

**Results:**

Nineteen studies with 1,287 hepatitis B-related cirrhotic patients were included. Most cirrhotic patients were compensated and achieved complete virological suppression when they stopped the antiviral therapy. The pooled proportions of virological relapse and clinical relapse after NAs discontinuation in cirrhotic patients were 55.23 (95% CI: 40.33–69.67) and 43.56% (95% CI: 26.13–61.85), respectively. HBsAg loss was observed in 56 of 500 (pooled proportion = 13.68%, 95% CI: 5.82–24.18) cirrhotic patients. And the pooled proportions of HCC development, hepatic decompensation and overall mortality were 8.76 (95% CI: 2.25–18.95), 3.63 (95% CI: 1.31–7.03), and 0.85% (95% CI: 0.35–1.57), respectively, after NAs discontinuation in cirrhotic patients.

**Conclusion:**

In hepatitis B-related compensated cirrhosis, who have achieved complete virological suppression, discontinuation of oral antivirals still carries a high relapse rate, but the incidence of adverse events is generally low and controlled during follow-up of at least 12 months. Of attention is that discontinuation of NAs can achieve a high rate of HBsAg seroclearance. This study may be helpful in the management of NAs in cirrhotic patients.

**Systematic review registration:**

http://www.crd.york.ac.uk/PROSPERO, identifier: CRD42020170103.

## Introduction

Liver cirrhosis is widely prevalent worldwide and associated with high morbidity and mortality (1, 2). In the past two decades, viral hepatitis has surpassed tuberculosis and diarrheal diseases as a major cause of mortality globally (3). About 2 million deaths worldwide annually are attributable to liver disease: 1 million due to cirrhosis and 1 million due to viral hepatitis and hepatocellular carcinoma, causing cirrhosis the 11th most common cause of death globally (4, 5). The incidence rate of cirrhosis in chronic hepatitis B patients is 1–2% (6–8). At present, antiviral therapy is primarily used to inhibit HBV replication to prevent the development of liver cirrhosis, hepatocellular carcinoma, and other liver-related complications.

Although there were reasonable concerns because of increasing rates of viral resistance with the use of the first-generation and second-generation oral antivirals, particularly lamivudine, the probability of HBV resistance is negligible (0–1%) during long-term monotherapy with the currently recommended oral agents entecavir and tenofovir (9–11). Entecavir or tenofovir monotherapy has been shown to achieve inhibition of HBV replication in almost all adherent patients. These drugs ameliorate liver fibrosis and can reverse cirrhosis in the majority of patients (12, 13). In addition, they eventually improve the morbidity and mortality of treated patients (9, 10). All NAs have an excellent tolerance and a good safety profile, but they cannot achieve HBV clearence or at least HBsAg loss in the vast majority of cases (9, 10). Therefore they should be given for very long periods, perhaps indefinitely, especially in cirrhotic patients (9, 10). The need for long-term NAs therapy raises safety issues for some patients (e.g., elderly patients with comorbidities like diabetes and renal insufficiency) and family planning issues in patients of reproductive age along with increases in treatment costs. For these reasons, many physicians treating CHB patients with NAs for years have become interested in investigating the need for continuation as well as the safety of therapy withdrawal. Several groups have started discontinuing NAs after a variable duration of treatment, but no definite conclusion has been reached yet (14).

Recent studies have found that cessation of oral antiviral therapy in patients with cirrhosis is also feasible and hepatic decompensation developed in only 1 of the 165 patients with cirrhosis included in 18 studies (15). However, another study showed that 2 of the 27 patients with cirrhosis developed hepatic decompensation off-therapy (16). Therefore, more research is needed to explore the clinical outcomes of stopping long-term NAs therapy in patients with cirrhosis.

Thus, the purpose of this systematic review is to evaluate existing data on clinical outcomes of NAs discontinuation in hepatitis B-related cirrhosis. This systematic review was prepared according to widely accepted recommendations (17).

## Materials and methods

This systematic review was performed according to the MOOSE and reported in accordance with PRISMA statement (17, 18). The protocol was registered at PROSPERO (CRD42020170103, http://www.crd.york.ac.uk/PROSPERO).

### Search strategy and article screening

The Medline and EMBASE databases were searched from inception up to May 2022 with the following terms: “Antiviral Agents or Lamivudine or entecavir or adefovir or telbivudine or tenofovir or nucleoside or nucleotide” AND “Hepatitis B or hepatitis B or HBV” AND “liver cirrhosis or Hepatic Cirrhosis or Cirrhosis, Hepatic or Cirrhosis, Liver” AND “discontinuation or withdrawal or end” (The detailed search strategy was described in Supplementary Tables 1, 2). Besides, we reviewed the references in identifying projects for further potential studies. Two reviewers independently screened the titles and abstracts of all retrieved records to find potentially appropriate studies, and then by reading the full text they evaluated the remaining records to identify studies suitable for data synthesis. Any disagreement was resolved by consensus or arbitrator.

### Inclusion criteria

We finally included original articles that met the following criteria:

1. They were observational studies (cohort or case-control) or randomized trials.
2. Participants in the study included adult patients with CHB including cirrhosis who discontinued oral antiviral(s) in virological remission defined by undetectable HBV DNA.
3. The duration of therapy was >12 months and the off-therapy follow-up was at least 12 months.

### Exclusion criteria

1. Non-English published studies.
2. Conference abstracts, reviews, comments, opinions, letters, and editorials.
3. Case reports, biochemical and experimental studies.
4. Patients with a history of HCC, liver transplantation, chemotherapy, or any systemic therapy with anti-viral agents, immunomodulators, cytotoxic agents or corticosteroids within 6 months.

### Information extraction and quality assessment

Basic information about each included study was extracted by two reviewers independently. The following information of the included trials was extracted: first author, time of publication, region, type of study design, number of cirrhotic participants, the methods used to diagnose cirrhosis, the types of NAs, the stop criteria, duration of follow-up after cession and the clinical outcomes. Clinical outcomes included virological relapse (VR, VR was defined as detectable serum HBV DNA), clinical relapse (CR, CR was defined as ALT >1.5 × ULN), HBsAg loss, HCC development, hepatic decompensation, and overall mortality in cirrhotic patients. Two investigators independently assessed the Newcastle–Ottawa scales (NOS) for all the studies except for abstracts. The reason for not conducting a bias assessment for abstracts was a lack of sufficient information. If there was discordance between the reviewers, a third reviewer was consulted to assist with the decision.

### Data extraction and statistical processing

Summary data of studies included in the systematic review were presented as reported in their original studies using frequencies with percentages for categorical variables, median with interquartile range (IQR) and mean with standard deviation for continuous variables. Estimation of pooled proportions and the corresponding 95% Clopper–Wilson confidence interval (CI) were calculated using transformed proportions using Freedman-Tukey double arcsine transformation to include studies with zero events and reported by using back transformation.

Heterogeneity between studies included was assessed using the Cochran's Q statistic (*Q*, Chi-squared test). Heterogeneity was present if *Q* > *df* (degree of freedom) with *P* ≤ 0.10. Quantification of heterogeneity was obtained using the inconsistency index (*I*^2^ statistic). *I*^2^-value of 0–29.99, 30–59.99, 60–74.99, and 75–100% indicate low, moderate, substantial, and considerable heterogeneity, respectively. Funnel plots were used to assess publication bias graphically and Egger's test was used for testing the asymmetry of the funnel plot. Sensitivity analysis was conducted by excluding studies with high bias. Newcastle–Ottawa Scales (NOS) was used to determine the quality of each study and poor quality is defined with a total maximum score of NOS ≤ 5.

Data analyses were conducted by R version 3.6.2 and all *P*-values were two-tailed and regarded as significant when *P* < 0.05 unless otherwise indicated.

## Results

**Figure 1:**
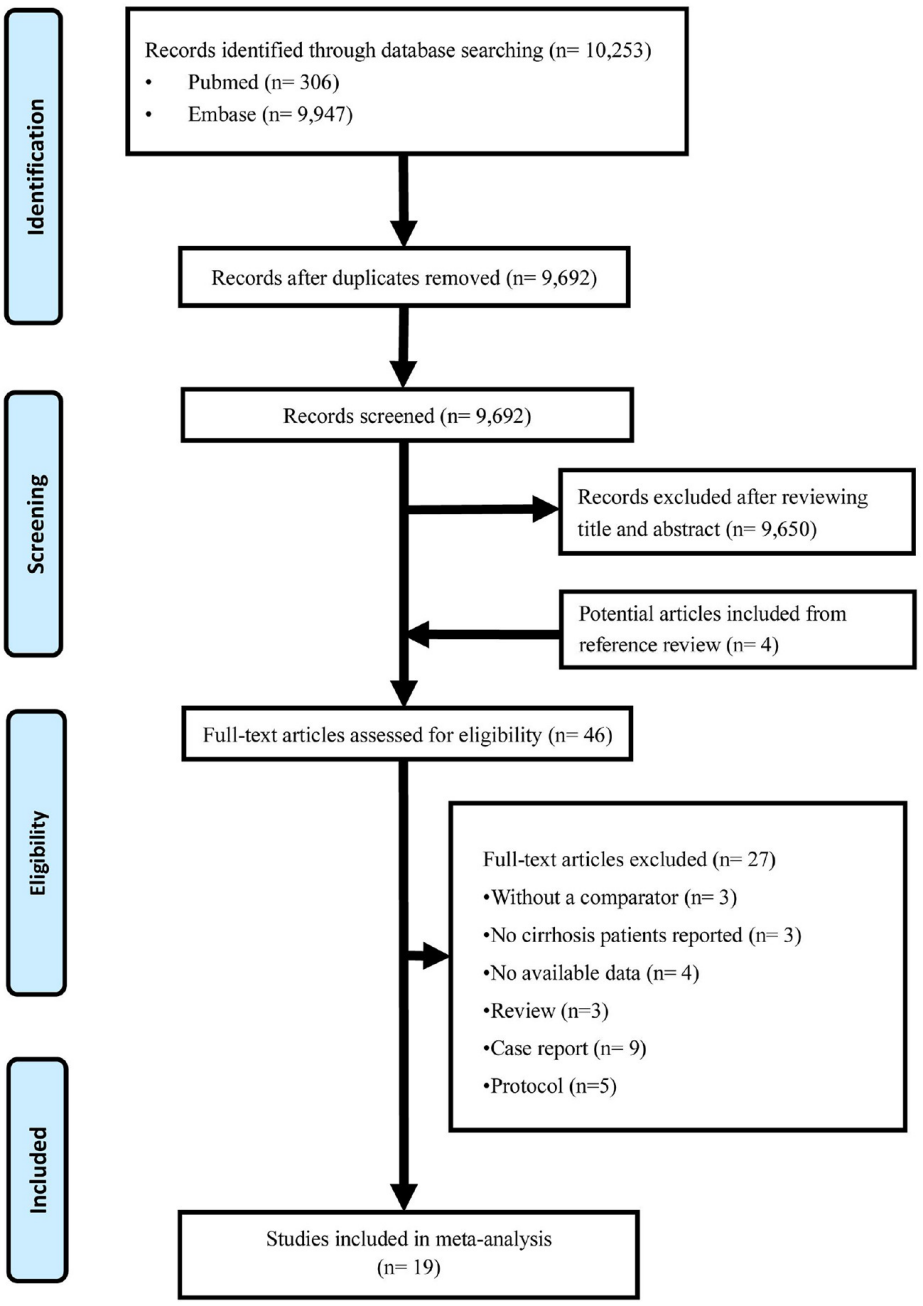
Flow diagram of study selection.

We retrieved 10,253 records from databases search and assessed 9,692 records after deleting the duplication, and finally, 19 original articles (19–37) were enrolled for data synthesis, as is shown in Figure 1. This systematic review finally yielded information on a total of 1,287 cases of chronic hepatitis B cirrhosis.

### Basic information and quality assessment

The basic information of the included studies is shown in Table 1. Thirteen of the included studies were retrospective (19–21, 23, 25–27, 32–37), two prospective (24, 31), and four retrospective-prospective studies (22, 28–30) with publication years between 2004 and 2022. Regarding race, sixteen of the subjects were of Asian origin (19–22, 24, 27–37), two were multicenter studies (23, 25), and one was unknown (26). For the diagnosis of cirrhosis, most studies relied on ultrasound or histologic findings; some used liver biopsy and other ancillary clinical features such as splenomegaly, portal hypertension, and endoscopic varices to aid in diagnosing cirrhosis. Concerning the stop NAs criteria, eleven studies were discontinued according to global guidelines or guideline protocols for chronic hepatitis B (20–22, 27, 28, 30–34, 37). Two studies were unknown, and discontinuation criteria for the remaining studies are shown in the Table 1 and Supplementary Table 4. Ninety-four cirrhotic patients in one study had a history of hepatic decompensation (19), while the rest have no history of decompensation. All included patients were HBsAg-positive at the time of discontinuation. All patients were followed up for at least 12 months after discontinuation. Quality assessment was shown in Supplementary Table 3. Among these 19 eligible studies, the scores of Newcastle-Ottawa quality were ranging from 6 to 9. All studies scored six stars or more. Moreover, most studies were adjusted or matched for the following confounders: age (15/19), and gender (14/19) (Table 1).

**Table 1:** Characteristics and quality of studies included in the systematic review.

**Author**	**Year**	**Area**	**Study design**	**Cirrhosis patients (*n*)**	**Cirrhosis diagnose**	**Duration of follow-up after cession (months)**	**NAs use**	**Stop criteria**	**NOS scores**	**Adjusted factors**
Fung et al. (24)	2004	USA	PC^†^	7	Biopsy	>12	LAM	Undetectable HBV DNA by PCR and normal ALT on at least three consecutive occasions during the second year of therapy	7	1, 2
Shinkai et al. (35)	2006	Japan	RC^‡^	4	Ultrasound	41	LAM	HBV DNA < 3.7 LGE/ml during at least six consecutive months	8	1, 2
Yeh et al. (37)	2009	Taiwan	RC	11	Biopsy or ultrasound diagnosis plus the presence of esophageal varices	72	LAM	ACT-HBV Asia-Pacific steering committee	8	1, 2
Jung et al. (32)	2011	Korea	RC	4	Histological or ultrasonographic findings plus splenomegaly (>10 cm) with a low platelet count (<100,000/mm^3^)	30	ADV	APASL 2008	7	1, 2
Jeng et al. (30)	2013	Taiwan	RC-PC	39	Histologic or clinical evidence	12	ETV	APASL 2008	7	2
Kim et al. (34)	2013	Korea	RC	9	Ultrasound	26.2	ETV/LAM/ADV	APASL 2008	7	1, 2
Chen et al. (20)	2014	Taiwan	RC	85	NR^§^	36	LAM/ETV/TEB	APASL 2012	7	–
Chi et al. (23)	2014	Multi-center	RC	27	NR	19.4	NR	HBeAg-negative,HBV-DNA <200 IU/mL	6	1
Sohn et al. (36)	2014	Korea	RC	44	Biopsy or clinical findings indicating the presence of portal hypertension	22	ETV/LAM/clevudine	For HBeAg positive patients, at least 6 months of consolidation therapy after achieving HBeAg loss and complete VR^¶^; for HBeAg-negative patients, complete VR maintained for at least 12 months by consolidation therapy	8	1, 2
Chen et al. (21)	2015	Taiwan	RC	97	NR	120	LAM/ETV	APASL 2008 or 2012	8	1
Chang et al. (19)	2015	Taiwan	RC	94	Histologic findings or ultrasonographic evidence	89.1	LAM	NR	9	1, 2
Chen et al. (22)	2015	Taiwan	RC–PC	164	Histological findings or ultrasonography or CT	54.6	ETV	APASL 2012	9	1, 2
Jung et al. (31)	2016	Korea	PC	21	Biopsy or ultrasound or portal hypertension-related complications evidence	12	ETV/LAM	APASL 2012	8	1, 2
Jeng et al. (29)	2016	Taiwan	RC–PC	34	Histologic findings or ultrasonographic evidence	33.3	TDF	Undetectable HBV-DNA on three occasions at least 6 months	8	1, 2
Hung et al. (27)	2017	Taiwan	RC	73	Ultrasound	72	LAM/ETV/TEB	APASL 2012	8	1, 2
Kang et al. (33)	2017	Korea	RC	29	NR	106	LAM	AASLD 2009, KASL 2011, APASL 2012	8	1, 2
Jeng et al. (28)	2018	Taiwan	RC–PC	308	Pathology or ultrasonography plus splenomegaly/endoscopic varices	154.8	ETV/TDF	APASL 2008	8	–
Hsu et al. (26)	2019	NR	RC	53	Ultrasound	38	NR	NR	6	–
Hirode et al. (25)	2022	Multi-center	RC	184	Histologic findings or ultrasonographic evidence	39.4	ETV/TDF/other	HBeAg-negative with at least 6 mo of undetectable HBV DNA/the discretion of the treating physician/patient's own initiative/APASL guidelines	9	1, 2, 3

Adjusted factors: 1, age; 2, sex; 3, Nonwhite.

† PC, prospective cochort;

‡ RC, retrospective cohort;

§ NR, not reported;

¶ VR, virological remission.

### Pooled proportion of VR after NAs discontinuation

**Figure 2:**
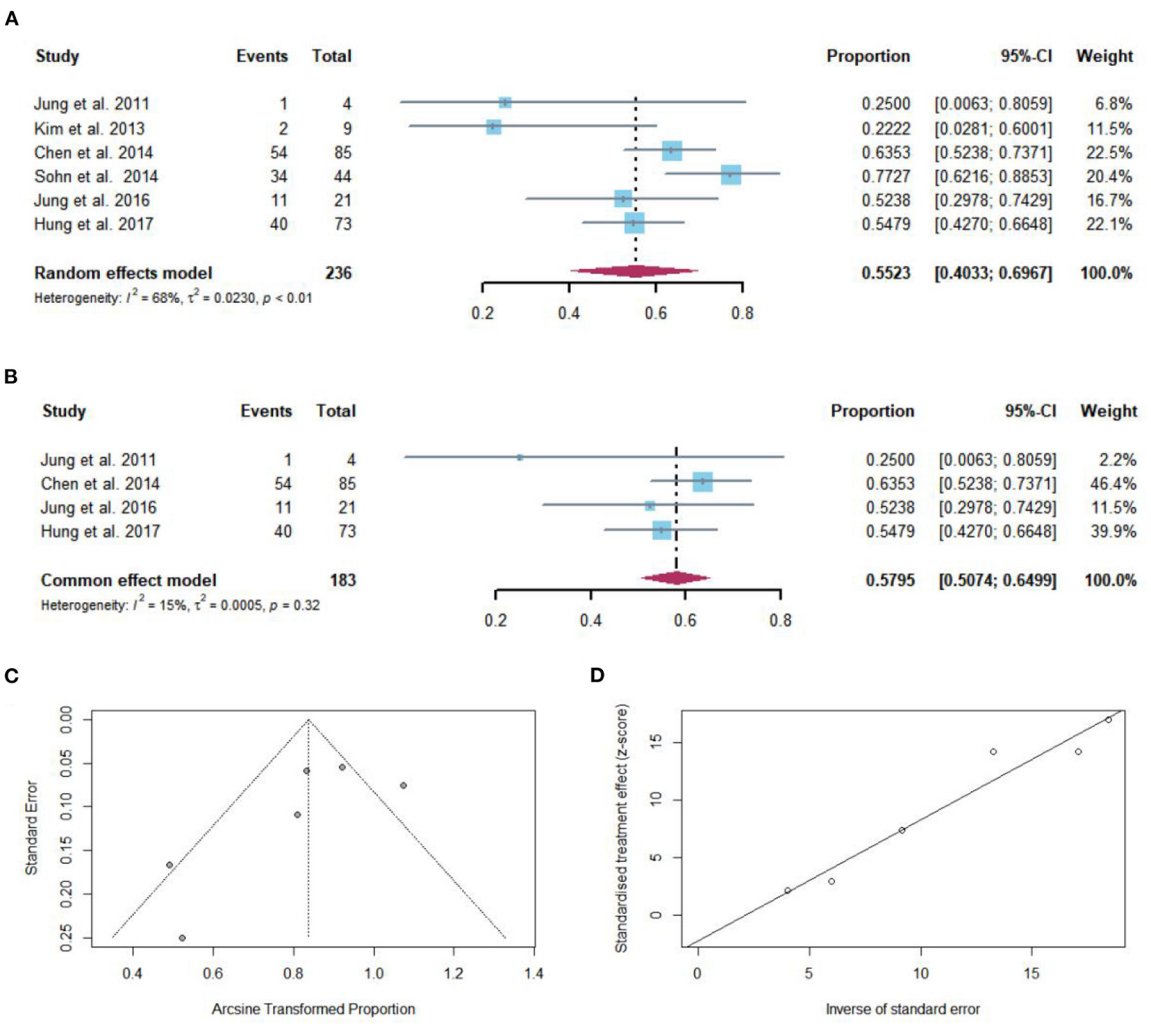
**(A)** Individual estimate and pooled proportion of VR in cirrhotic patients after NAs discontinuation. **(B)** Sensitivity analysis of pooled proportion of VR in cirrhotic patients after NAs discontinuation excluding studies with high bias. **(C)** Funnel plot. **(D)** Egger's test.

Regardless of the duration of follow-up, VR after NAs discontinuation was reported in 142 of 236 patients with cirrhosis. The pooled proportion of VR (*n* = 6 studies) after NAs discontinuation was 55.23% (95% CI: 40.33–69.67) using a random-effects model (Figure 2A). Substantial heterogeneity was found among these studies (*I*^2^ = 68%; *Q* = 15.67, *df* = 5, *P* < 0.01) without publication bias (Figures 2C,D). In sensitivity analysis exclusion of studies with high bias, resulted in a small increase of the estimated pooled proportion (50.74–64.99%) of VR in cirrhotic patients who stopped NAs (Figure 2B).

**Figure 3:**
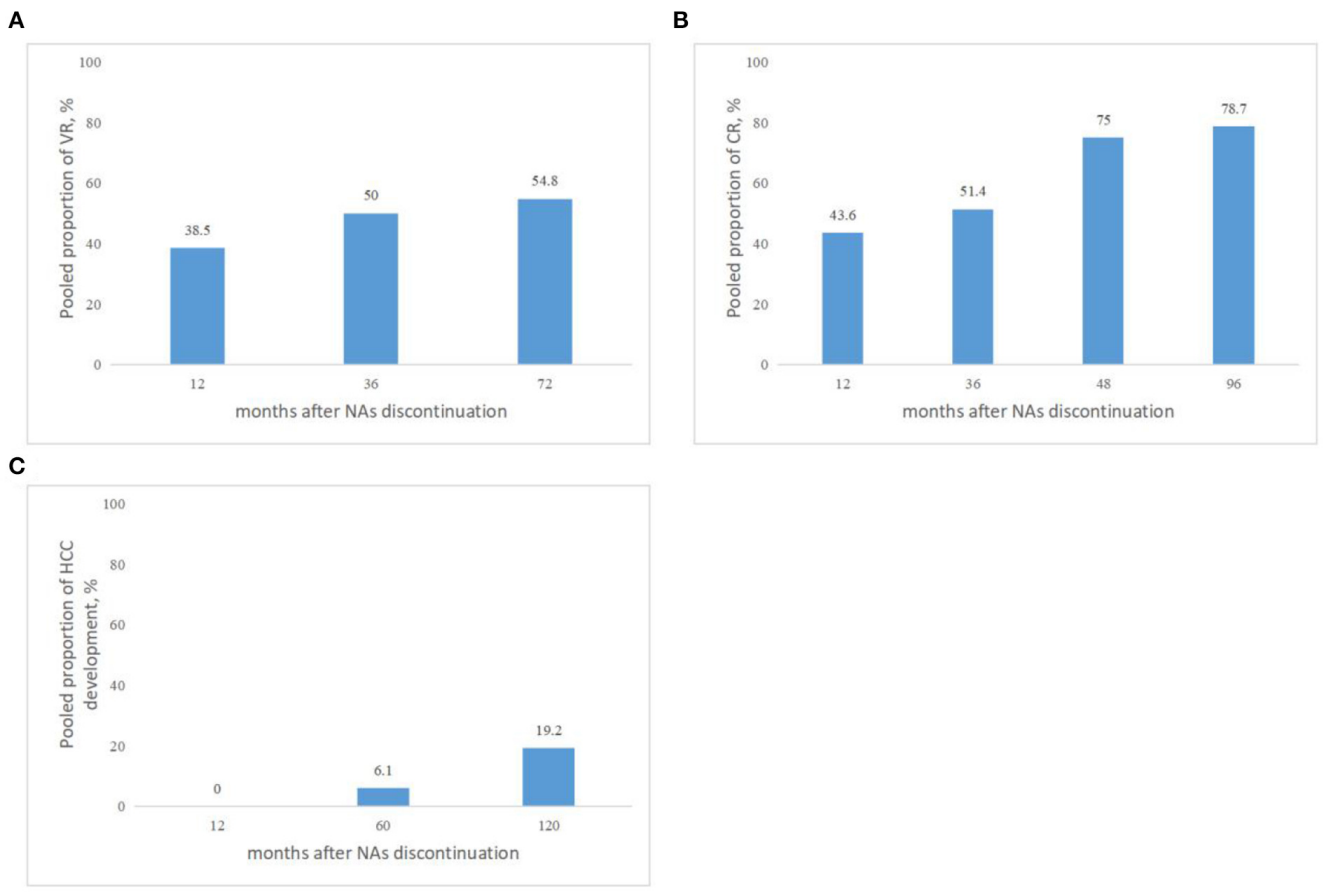
**(A)** Pooled proportions of VR in cirrhotic patients after NAs discontinuation. VR was defined by serum HBV DNA levels >2,000 IU/mL. **(B)** Pooled proportions of CR in cirrhotic patients after NAs discontinuation. CR was defined as ALT > 2 × ULN. **(C)** Pooled proportions of HCC development in cirrhotic patients after NAs discontinuation.

Taking into consideration the duration of follow-up, while defined VR by serum HBV DNA levels >2,000 IU/mL, the pooled proportions (95% CI) of VR were 38.5 (9.1–68), 50 (13.9–86), 54.8% (42.7–66.5) at 12, 36, and 72 months, respectively, after NAs cessation (Figure 3A).

### Pooled proportion of CR after NAs discontinuation

**Figure 4:**
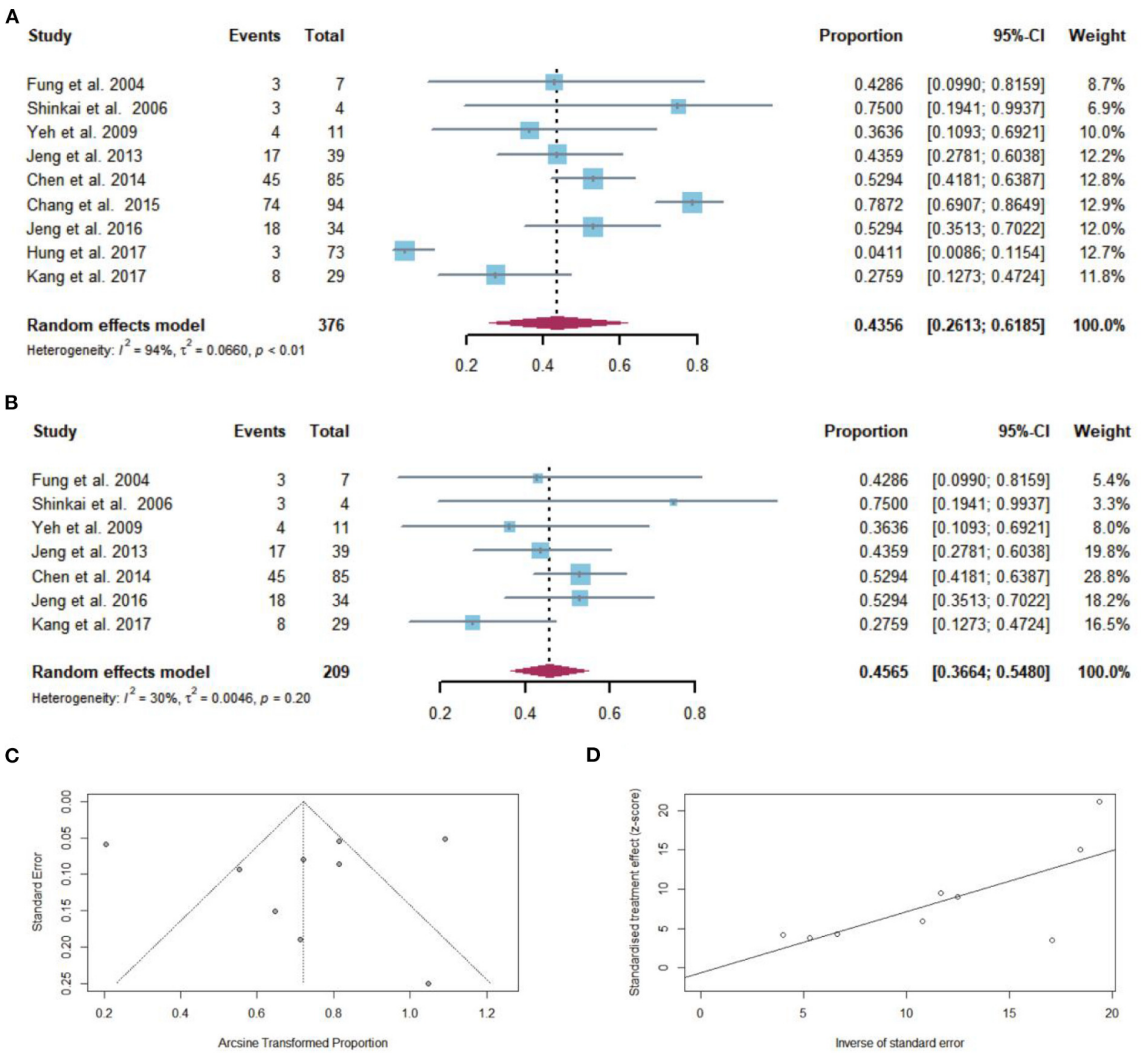
**(A)** Individual estimate and pooled proportion of CR in cirrhotic patients after NAs discontinuation. **(B)** Sensitivity analysis of pooled proportion of CR in cirrhotic patients after NAs discontinuation excluding studies with high bias. **(C)** Funnel plot. **(D)** Egger's test.

Regardless of the duration of follow-up, CR after NAs discontinuation was reported in 175 of the 376 patients with cirrhosis. The pooled proportion of CR (*n* = 9 studies) after NAs discontinuation was 43.56% (95% CI: 26.13–61.85) using a random-effects model (Figure 4A). Considerable heterogeneity was found among these studies (*I*^2^ = 94%; *Q* = 138.84, *df* = 8, *P* < 0.01) without publication bias (Figures 4C,D). In sensitivity analysis exclusion of studies with high bias, resulted in a small increase of the estimated pooled proportion (36.64–54.80%) of CR in cirrhotic patients who stopped NAs (Figure 4B).

Taking into consideration the duration of follow-up, while CR was defined as ALT > 2 × ULN after NAs discontinuation, the pooled proportions (95% CI) of CR were 43.6 (27.8–60.4), 51.4 (42.9–60), 75 (19.4–99.4), 78.7% (69.1–86.5) at 12, 36, 48, and 96 months, respectively, after NAs cessation (Figure 3B).

### Pooled proportion of HBsAg loss after NAs discontinuation

**Figure 5:**
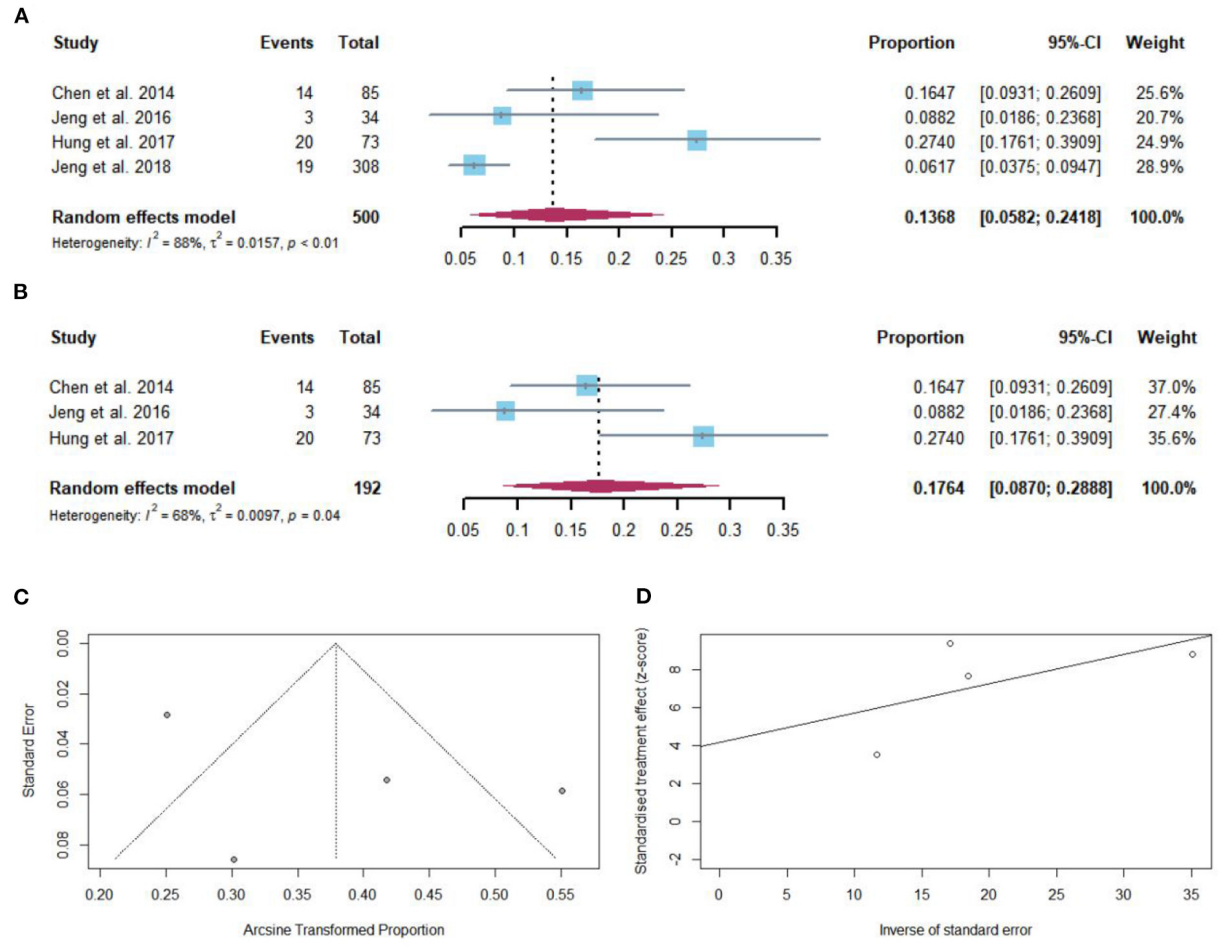
**(A)** Individual estimates and pooled proportion of HBsAg loss in cirrhotic patients after NAs discontinuation. **(B)** Sensitivity analysis of pooled proportion of HBsAg loss in cirrhotic patients after NAs discontinuation excluding studies with high bias. **(C)** Funnel plot. **(D)** Egger's test.

Fifty-six of 500 cirrhotic patients achieved HBsAg loss after NAs discontinuation. The pooled proportion of HBsAg seroclearance (*n* = 4 studies) after NAs discontinuation was 13.68% (95% CI: 5.82–24.18) using a random-effects model (Figure 5A). Considerable heterogeneity was found among these studies (*I*^2^ = 88%; *Q* = 24.65, *df* = 3, *P* < 0.01) without publication bias (Figures 5C,D). In sensitivity analysis, after the removal of studies with high bias, there was an increase in the overall pooled proportion of HBsAg loss (8.70–28.88%) in cirrhotic patients who stopped NAs (Figure 5B).

### Pooled proportion of HCC development after NAs discontinuation

**Figure 6:**
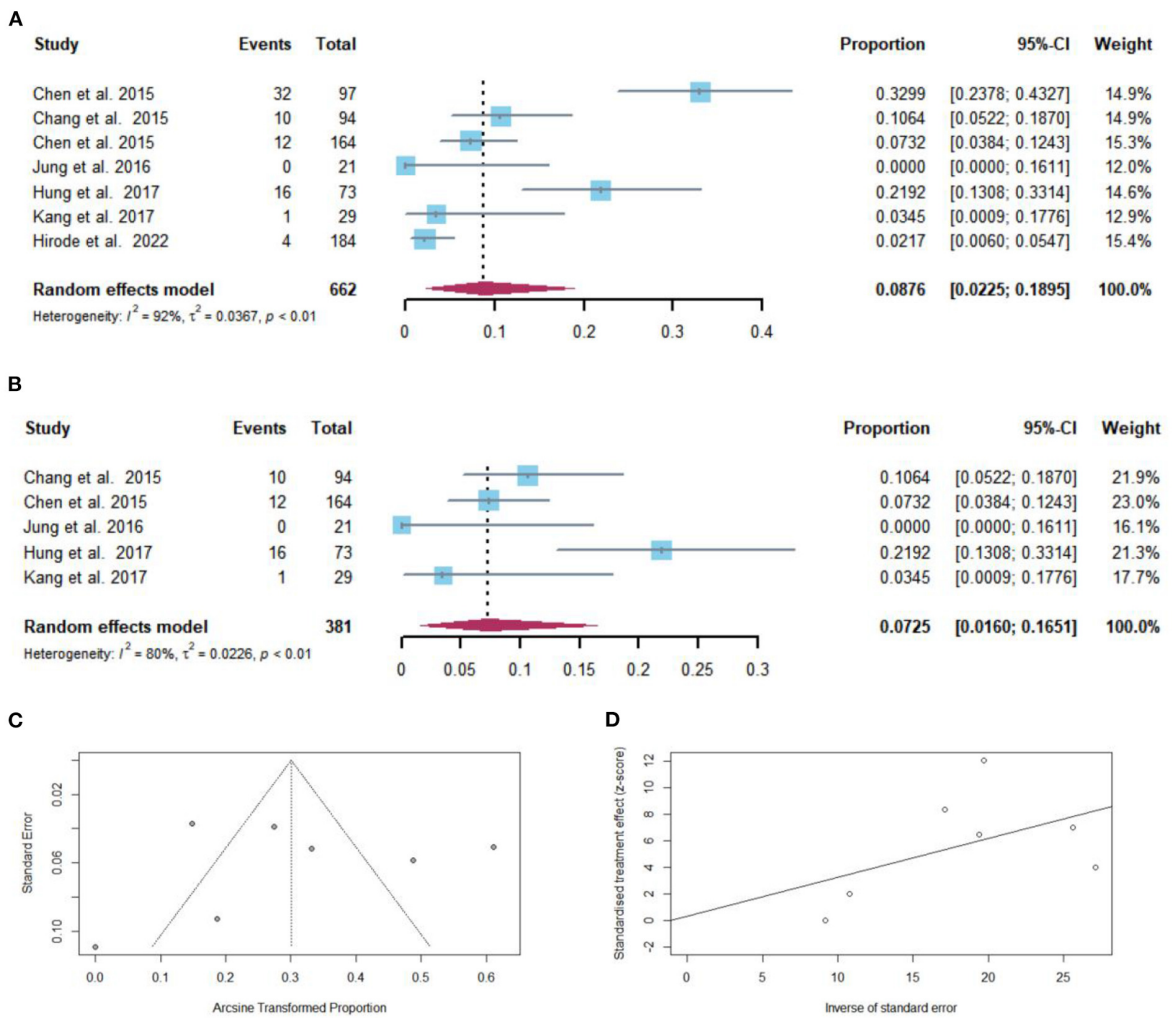
**(A)** Individual estimates and pooled proportion of HCC development in cirrhotic patients after NAs discontinuation. **(B)** Sensitivity analysis of pooled proportion of HCC development in cirrhotic patients after NAs discontinuation excluding studies with high bias. **(C)** Funnel plot. **(D)** Egger's test.

After NAs discontinuation, HCC developed in 75 of 662 cirrhotic patients. The pooled proportion of HCC development (*n* = 7 studies) after NAs discontinuation was 8.76% (95% CI: 2.25–18.95) using a random-effects model (Figure 6A). Considerable heterogeneity was found among these studies (*I*^2^ = 92%; *Q* = 74.70, *df* = 6, *P* < 0.01) without publication bias (Figures 6C,D). In sensitivity analysis, after the removal of studies with high bias, there was a decrease in the overall pooled proportion of HCC development (1.60–16.51%) in cirrhotic patients who stopped NAs (Figure 6B).

Taking into consideration the duration of follow-up, the pooled proportions (95% CI) of HCC development were 0 (0–16.1), 6.1 (1.2–10.9), 19.2% (2.1–36.3) at 12, 60, and 120 months, respectively, after NAs cessation (Figure 3C).

### Pooled proportion of hepatic decompensation events after NAs discontinuation

**Figure 7:**
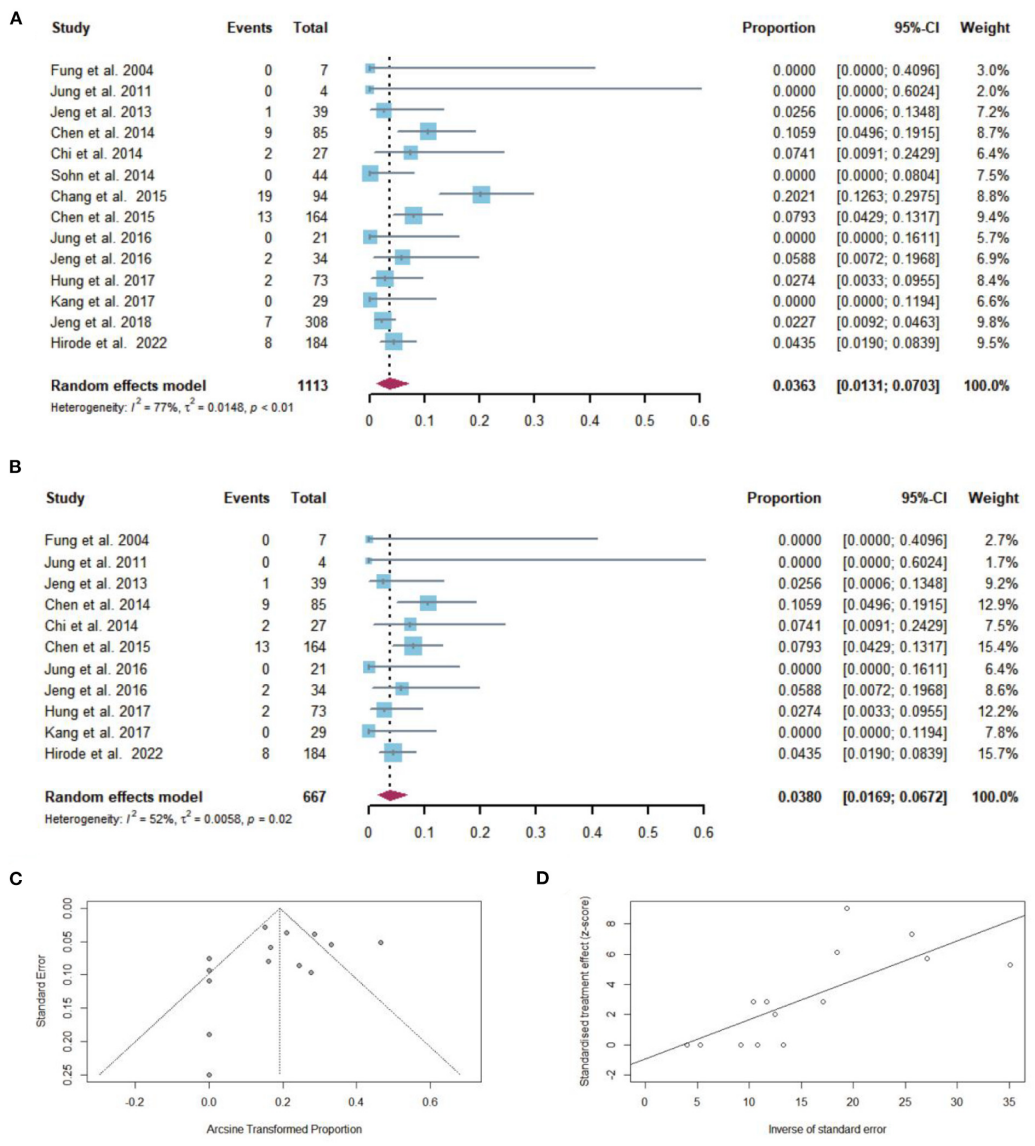
**(A)** Individual estimates and pooled proportion of hepatic decompensation in cirrhotic patients after NAs discontinuation. **(B)** Sensitivity analysis of pooled proportion of hepatic decompensation in cirrhotic patients after NAs discontinuation excluding studies with high bias. **(C)** Funnel plot. **(D)** Egger's test.

Sixty-three of 1,113 patients with cirrhosis developed hepatic decompensation after NAs discontinuation, including one study that had a history of hepatic decompensation. The pooled proportion of hepatic decompensation (*n* = 14 studies) after NAs discontinuation was 3.63% (95% CI: 1.31–7.03) using a random-effects model (Figure 7A). Considerable heterogeneity was found among these studies (*I*^2^ = 77%; *Q* = 57.70, *df* = 13, *P* < 0.01) without publication bias (Figures 7C,D). In sensitivity analysis, there was minimal effect on pooled proportion of hepatic decompensation in cirrhotic patients who stopped NAs (Figure 7B).

After excluding the study that had a history of hepatic decompensation, the pooled proportion of hepatic decompensation after NAs discontinuation was 2.95% (95% CI: 1.20–5.45). In two studies in which cirrhotic patients took timely retreatment after relapse, the pooled proportion of hepatic decompensated was 0%.

### Pooled proportion of overall mortality after NAs discontinuation

**Figure 8:**
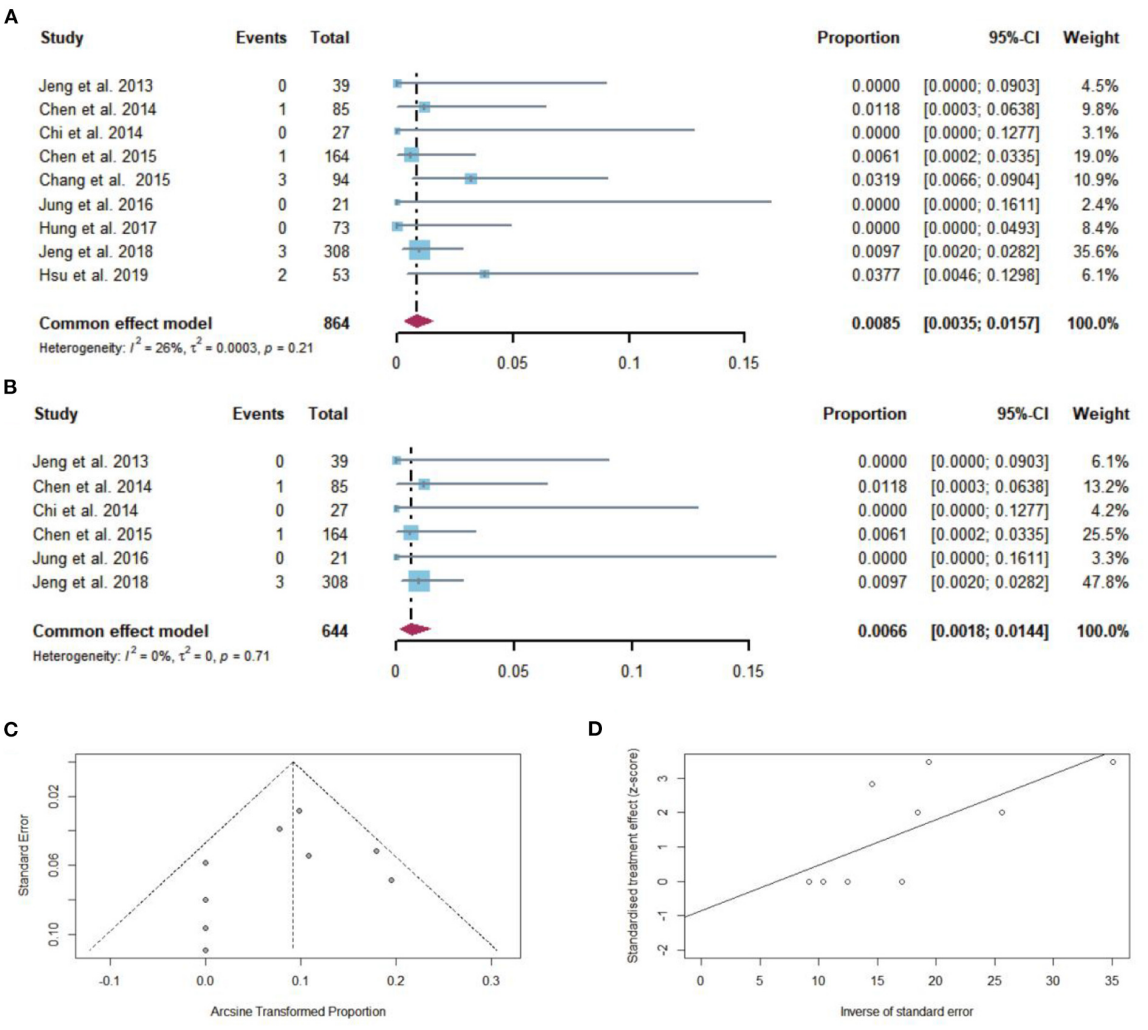
**(A)** Individual estimates and pooled proportion of overall mortality in cirrhotic patients after NAs discontinuation. **(B)** Sensitivity analysis of pooled proportion of overall mortality in cirrhotic patients after NAs discontinuation excluding studies with high bias. **(C)** Funnel plot. **(D)** Egger's test.

Among 864 cirrhotic patients, 10 died after NAs discontinuation. The pooled proportion of overall mortality (*n* = 9 studies) after NAs discontinuation was 0.85% (95% CI: 0.35–1.57) using a fixed-effects model (Figure 8A). Low heterogeneity was found among these studies (*I*^2^ = 26%; *Q* = 10.85, *df* = 8, *P* = 0.21) without publication bias (Figures 8C,D). In sensitivity analysis, after the removal of studies with high bias, there was a small decrease in the overall pooled proportion of overall mortality (0.18–1.44%) in cirrhotic patients who stopped NAs (Figure 8B).

After excluding the study that had a history of hepatic decompensation, the pooled proportion of overall mortality after NAs discontinuation was 0.67% (95% CI: 0.21–1.36).

## Discussion

The global guidelines have not fully harmonized the criteria for discontinuing NAs in patients with hepatitis B-related cirrhosis. The APASL guidelines suggest that patients with cirrhosis can be considered for discontinuation of NAs with close follow-up (38), while both EASL and AASLD guidelines suggest indefinite treatment (The detailed content was described in Supplementary Table 4). High-risk events such as liver failure, HCC, and death that may be caused by virological relapse after cessation of NAs and the risk of drug resistance after restarting antiviral therapy are currently the main concerns after discontinuation of antiviral therapy (39, 40).

However, some studies showed that in patients with cirrhosis, the main risk of HCC could not be eliminated with prolonged NAs therapy (22, 27, 28, 41). Meanwhile, one study showed that NAs treatment leads to dynamic histologic improvement only within the first 4 years of therapy (42). The benefits of long-term NAs may be overestimated in cirrhotic patients, especially in those who have achieved complete virological suppression. Thus, it is reasonable to question if indefinite NAs treatment is mandatory in cirrhotic patients. Therefore, our study systematically reviews the clinical outcomes of discontinuing NAs in CHB patients with cirrhosis.

All included patients in our study were compensated cirrhotic patients, except 94 cirrhotic patients in one study who had a history of hepatic decompensation. All patients were followed up for at least 12 months after discontinuation. Therefore, our study may be the first report of long-term follow-up clinical outcomes after the discontinuation of NAs in compensated cirrhotic patients.

Regarding the development of HCC, our study found that the rate of HCC after NAs discontinuation in cirrhotic patients was 8.76% (95% CI: 2.25–18.95). Some systematic reviews suggest that the risk of HCC in cirrhotic patients on long-term antiviral therapy is about 10–11% (43, 44). It suggests that HCC may not significantly develop after NAs discontinuation. Several studies have shown that the development of HCC in cirrhotic patients is not significantly associated with NAs discontinuation, and NAs may not prevent HCC in those who have developed cirrhosis, especially in long-term follow-ups (22, 27, 28, 41). The persistence of cccDNA and integrated HBV genome in CHB patients constitutes a higher risk of HCC, especially in those with cirrhosis. Also, in patients with cirrhosis, the main risk of HCC will no longer be caused by viremia but by the complex immune and pathological mechanisms initiated by cirrhosis (45–47), resulting in that NAs may not effectively reduce the risk of HCC in those who have developed cirrhosis.

The risk of hepatic decompensation and death is another reason why discontinuing NAs in cirrhosis is currently not generally supported. In our study, the incidence of hepatic decompensation after discontinuation of NAs in cirrhotic patients was 3.63%, and the overall mortality rate was only 0.85%. In those without a history of hepatic decompensation, both rates were lower, 2.95 and 0.67%, respectively. Among those who took timely retreatment after relapse, the incidence of hepatic decompensated was 0% (31, 36). Thus, discontinuing NAs in compensated cirrhotic patients who have achieved complete virologic suppression may not result in a high risk of decompensation and death. In a study with a mean follow-up of 6 ± 3 years, the risk of liver decompensation in cirrhotic patients on long-term NAs was 11%, with 5 and 10-year event-free survivals rates of 70.2 and 40.4%, respectively (48). This study indicated that long-term NAs improved adverse outcomes in CHB patients primarily by reducing the development of cirrhosis, and the effect of NAs was diminished in those who have already developed cirrhosis.

Sustained off-therapy HBsAg loss or < 0.05 IU/mL (with or without seroconversion to HBsAb) and undetectable HBV DNA (or < 10 IU/mL) is currently the ideal therapy endpoint for CHB patients. However, it was still difficult and unrealistic to achieve this goal during NAs treatment (39). The decline/seroclearance rate of HBsAg was significantly higher in patients stopping NAs therapy (39). In our study, the seroclearance of HBsAg in cirrhotic patients after discontinuation of NAs therapy was 13.68% (95% CI: 5.82–24.18). According to Hung et al. (27), study, in those who continued antiviral therapy, this rate was only 3.16%. Moreover, Hung et al. (27), study showed significant statistical difference in HBsAg loss in cirrhotic patients who discontinued or continued NAs. HBsAg seroclearance would effectively reduce the risk of long-term adverse outcomes (liver failure, HCC, death) in cirrhotic patients (49–52). Given the low rate of decompensated and mortality, HBsAg seroclearance achieved by NAs discontinuation seems worthwhile. HBsAg seroclearance after NAs discontinuation may be a desirable benefit, which may not be available for those on-therapy, to improve the clinical outcomes in cirrhotic patients.

Long-term NAs treatment for CHB is recommended in all regional guidelines, especially in cirrhosis. It is agreed that NAs can halt HBV replication, but it has to be controlled with host immunity. Our findings showed that in patients with compensated cirrhosis, who have achieved complete virological suppression, discontinuation of oral antivirals still carries a high relapse rate, but the incidence of adverse events is generally low and controlled. Nevertheless, patients with compensated cirrhosis had a reasonable chance of achieving HBsAg loss after discontinuation of NAs, suggesting that host immune control of the virus and clinical cure can be achieved with controlled risk. However, the relationship between the discontinuation of NAs and the immune system still needs a significant amount of research.

There are some limitations of this study: (1) Our study included mostly retrospective cohort studies, and the lack of direct controls with non-stop patients may weaken the persuasiveness of the results. We expect more prospective studies to validate our results in the future. (2) Due to incomplete data acquisition, we did not perform subgroup analyses of factors that may affect outcomes after discontinuation (e.g., gender, age, duration of NAs use and HBV genotype). (3) Since the diagnosis of cirrhosis is mostly derived from ultrasound, we did not perform an in-depth analysis of the impact of different stages of cirrhosis on outcomes. (4) The majority of the included participants were of Asian descent, which may have led to inadequate analysis of the results in terms of race. (5) Last but not least, our study only provides a guide and is exploratory in nature. The results of this study do not mean that patients with cirrhosis can be discontinued at will.

## Conclusion

In hepatitis B-related compensated cirrhosis, who have achieved complete virological suppression, discontinuation of oral antivirals still carries a high relapse rate, but the incidence of adverse events is generally low and controlled during follow-up of at least 12 months. Of attention is that discontinuation of NAs can achieve a high rate of HBsAg seroclearance. This study may be helpful in the management of NAs in cirrhotic patients.

## Data availability statement

The original contributions presented in the study are included in the article/Supplementary material, further inquiries can be directed to the corresponding author/s.

## Author contributions

JZ, YYa, and YYe: study conception and design. XZ and XC: data curation. XL, YYa, GC, and XC: formal analysis. GC: methodology and visualization. JZ: software. JZ and YYa: writing—original draft preparation. All authors contributed to the article and approved the submitted version.

## Funding

This work was supported by China National Science and Technology major projects 13th 5-year plan (No. 2018ZX10725505) and New Teacher Start-up Fund Project of Beijing University of Chinese Medicine (2022-JYB-XJSJJ-050). YYe and JZ have received research support, respectively.

## Conflict of interest

The authors declare that the research was conducted in the absence of any commercial or financial relationships that could be construed as a potential conflict of interest.

## Publisher's note

All claims expressed in this article are solely those of the authors and do not necessarily represent those of their affiliated organizations, or those of the publisher, the editors and the reviewers. Any product that may be evaluated in this article, or claim that may be made by its manufacturer, is not guaranteed or endorsed by the publisher.
