# Supplementary material for: Systematic review: Clinical outcomes of discontinuation of oral antivirals in hepatitis B-related liver cirrhosis
Source: Front Public Health. 2022 Nov 3;10:1037527. doi: 10.3389/fpubh.2022.1037527 (PMC9670108; doi:10.3389/fpubh.2022.1037527)
Supplement: Supplementary file 1 [file Data_Sheet_1.docx]

Supplementary Table 1. Search Strategy Used in PubMed, May, 2022

| Number | Search Items | Items Found |
| --- | --- | --- |
| 1 | "Antiviral Agents"[MeSH Terms] OR "Lamivudine"[All Fields] OR "entecavir"[All Fields] OR "adefovir"[All Fields] OR "telbivudine"[All Fields] OR "tenofovir"[All Fields] OR "nucleosid"[All Fields] OR "nucleosidation"[All Fields] OR "nucleosides"[MeSH Terms] OR "nucleosides"[All Fields] OR "nucleoside"[All Fields] OR "nucleosidic"[All Fields] OR "nucleotid"[All Fields] OR "nucleotides"[MeSH Terms] OR "nucleotides"[All Fields] OR "nucleotide"[All Fields] OR "nucleotidic"[All Fields] | 1,299,109 |
| 2 | "Hepatitis B"[MeSH Terms] OR "Hepatitis B"[MeSH Terms] OR "Hepatitis B"[All Fields] OR "HBV"[All Fields] | 110,903 |
| 3 | "liver cirrhosis"[MeSH Terms] OR "liver cirrhosis"[All Fields] OR "Hepatic Cirrhosis"[All Fields] OR "cirrhosis hepatic"[All Fields] OR "cirrhosis liver"[All Fields] | 112,276 |
| 4 | "withdraw"[All Fields] OR "withdrawal"[All Fields] OR "withdrawals"[All Fields] OR "withdrawing"[All Fields] OR "withdraws"[All Fields] OR "discontinuance"[All Fields] OR "discontinuances"[All Fields] OR "discontinuated"[All Fields] OR "discontinuation"[All Fields] OR "discontinuations"[All Fields] OR "discontinue"[All Fields] OR "discontinued"[All Fields] OR "discontinuer"[All Fields] OR "discontinuers"[All Fields] OR "discontinues"[All Fields] OR "discontinuing"[All Fields] OR "end"[All Fields] | 1,060,495 |
| 5 | Numbers 1–4 | 306 |

Supplementary Table 2. Search Strategy Used in Embase, May, 2022

| Number | Search Items | Items Found |
| --- | --- | --- |
| 1 | 'lamivudine'/exp OR 'adefovir dipivoxil'/exp OR 'antivirus agent'/exp OR 'telbivudine'/exp OR 'tenofovir'/exp OR 'entecavir'/exp OR adefovir OR entecavir OR telbivudine OR virucid* OR virustatic* OR tenofovir OR (nucleo?ide AND analogue*) OR therapy OR treatment | 13,468,105 |
| 2 | 'hepatitis b'/exp OR (hepatitis AND b) OR hbv | 473,049 |
| 3 | 'liver cirrhosis' OR 'hepatic cirrhosis' OR 'cirrhosis, hepatic' OR 'cirrhosis, liver' | 187,860 |
| 4 | 'withdrawal' OR 'discontinuation' OR 'end' | 1,665,590 |
| 5 | Numbers 1–4 | 9,947 |

#

# Supplementary Table 3: Newcastle-Ottawa Quality Assessments Scale (Newcastle-Ottawa Scale)

| Study | Year | Quality Indicators Form Newcastle-Ottawa Scale | | | | | | | | Scores |
| --- | --- | --- | --- | --- | --- | --- | --- | --- | --- | --- |
|  |  | 1 | 2 | 3 | 4 | 5 | 6 | 7 | 8 |  |
| Chen et al. [22] | 2015 | ★ | ★ | ★ | ★ | ★★ | ★ | ★ | - | 8 |
| Hung et al. [28] | 2017 | ★ | ★ | ★ | ★ | ★★ | ★ | ★ | - | 8 |
| Yeh et al. [38] | 2009 | ★ | ★ | ★ | ★ | ★★ | ★ | ★ | - | 8 |
| Chen et al. [21] | 2014 | ★ | ★ | ★ | ★ | ★ | ★ | ★ | - | 7 |
| Hirode et al. [26] | 2022 | ★ | ★ | ★ | ★ | ★★ | ★ | ★ | ★ | 9 |
| Chi et al. [24] | 2014 | ★ | ★ | ★ | ★ | ★ | ★ | - | - | 6 |
| Sohn et al. [37] | 2014 | ★ | ★ | ★ | ★ | ★★ | ★ | - | ★ | 8 |
| Jung et al. [32] | 2016 | ★ | ★ | ★ | ★ | ★★ | ★ | - | ★ | 8 |
| Chang et al. [20] | 2015 | ★ | ★ | ★ | ★ | ★★ | ★ | ★ | ★ | 9 |
| Shinkai et al. [36] | 2006 | ★ | ★ | ★ | ★ | ★★ | ★ | ★ | - | 8 |
| Kang et al. [34] | 2017 | ★ | ★ | ★ | ★ | ★★ | ★ | ★ | - | 8 |
| Fung et al. [25] | 2004 | ★ | ★ | ★ | ★ | ★★ | ★ | - | - | 7 |
| Jeng et al. [31] | 2013 | ★ | ★ | ★ | ★ | ★★ | ★ | - | - | 7 |
| Jeng et al. [30] | 2016 | ★ | ★ | ★ | ★ | ★★ | ★ | ★ | - | 8 |
| Jeng et al. [29] | 2018 | ★ | ★ | ★ | ★ | ★ | ★ | ★ | ★ | 8 |
| Chen et al. [23] | 2015 | ★ | ★ | ★ | ★ | ★★ | ★ | ★ | ★ | 9 |
| Hsu et al. [27] | 2019 | ★ | ★ | ★ | ★ | - | ★ | ★ | - | 6 |
| Kim et al. [35] | 2013 | ★ | ★ | ★ | ★ | ★★ | ★ | - | - | 7 |
| Jung et al. [33] | 2011 | ★ | ★ | ★ | ★ | ★★ | ★ | - | - | 7 |

★: Cohort Studies. 1.Representativeness of the exposed cohort/Definition of Cases; 2. Selection of the non-exposed cohort/Representativeness of Cases; 3. Ascertainment of exposure/Selection of Controls; 4. Outcome of interest not present at start of study/Definition of Controls; 5. Control for important factor or additional factor/Comparability; 6. Assessment of outcome/Assessment of exposure; 7. Follow-up long enough for outcomes to occur/Method of Ascertainment; 8. Adequacy of follow up of cohorts/Non-Response Rate.

Supplementary Table 4. International guidelines for commencement and cessation of NAs treatment in CHB patients

|  | When to start or restart | When to stop |
| --- | --- | --- |
| AASLD 2018 | 1. ALT >2 ULN and HBV DNA above 2,000 IU/mL (HBeAg negative) or above 20,000 IU/mL (HBeAg positive);  2. Consider severity of liver disease when ALT above the ULNs, but <2 ULN;  3. Consider additional factors when ALT <2 ULN and HBV DNA below thresholds | 1. HBeAg-positive CHB: seroconvert to anti-HBe on therapy discontinue NAs after a period of treatment consolidation; the period of consolidation therapy generally involves treatment for at least 12 months of persistently normal ALT levels and undetectable serum HBV DNA levels;  2. HBeAg-negative CHB: indefinite antiviral therapy unless HBsAg is lost. |
| EASL 2017 | 1. All patients with HBeAg-positive or -negative chronic hepatitis B, defined by HBV DNA > 2,000 IU/ml, ALT >ULN and/or at least moderate liver necroinflammation or fibrosis, should be treated;  2. Patients with HBV DNA >20,000 IU/ml and ALT >2×ULN should start treatment regardless of the degree of fibrosis. | 1. HBeAg-positive CHB: achieve stable HBeAg seroconversion and undetectable HBV DNA, and complete at least 12 months of consolidation therapy; or after confirmed HBsAg loss, with or without anti-HBs seroconversion;  2. HBeAg-negative CHB: achieved long-term (≥3 years) virological suppression under NAs may be considered if close post-NA monitoring can be guaranteed; or after confirmed HBsAg loss. |
| APASL 2015 | 1. HBeAg-positive CHB: (1) HBV DNA ≥20 000 IU/mL and ALT≥2×ULN; (2) HBV DNA ≥20 000 IU/mL or <20 000 IU/mL with any ALT, if evidence shows moderate/severe inflammation/fibrosis  2. HBeAg-negative CHB: (1) HBV DNA ≥2000 IU/mL and ALT≥2×ULN; (2) HBV DNA ≥2000 IU/mL or <2000 IU/mL with any ALT, if evidence shows moderate/severe inflammation/fibrosis. | 1. HBeAg-positive CHB: after at least 1 year, but preferably after 3 years of additional therapy after HBeAg seroconversion with undetectable HBV DNA by PCR and persistently normal ALT levels;  2. HBeAg-negative CHB: (1) after HBsAg loss following either anti-HBs seroconversion or at least 12 months of a post-HBsAg clearance consolidation period, or (2) after treatment for at least 2 years with undetectable HBV DNA documented on three separate occasions, 6 months apart. |
| APASL 2012 | Chronic HBV-infected patients with  ALT >2 times ULN, and HBV DNA >10^4^ IU/mL if HBeAg positive and >10^3^ IU/mL if HBeAg-negative as well as patients with advanced fibrosis or cirrhosis with any ALT level should be considered for treatment. | 1. In HBeAg-positive patients, treatment can be stopped when HBeAg seroconversion with undetectable HBV DNA has been maintained for at least 12 months. 2. In HBeAg-negative patients, it is not clear how long treatment should be continued if HBsAg remains positive, but treatment discontinuation can be considered if patients have been treated for at least 2 years with undetectable HBV DNA documented on three separate occasions 6 months apart. |
| APASL 2008 | Chronic HBV-infected patients with  ALT >2 times ULN and HBV-DNA >10^4^ IU/ml (10^5^ copies/ml) if HBeAg positive or >10^3^ IU/ml (10^4^ copies/ml) if HBeAg negative should be considered for treatment | 1. In HBeAg-positive patients, treatment can be stopped when HBeAg seroconversion with undetectable HBV-DNA has been documented on 2 separate occasions at least 6 months apart. 2. In HBeAg-negative patients, it is not   clear how long this treatment should be continued, but treatment discontinuation can be considered if undetectable HBV-DNA has been documented on three separate occasions 6 months apart. |
| WHO 2015 | All persons with cirrhosis based on clinical evidence (or APRI score >2 in adults) require lifelong treatment with nucleos(t)ide analogues (NAs), and should not discontinue antiviral therapy because of the risk of reactivation, which can cause severe acute-on-chronic liver injury. | 1.persons without clinical evidence of cirrhosis (or based on APRI score<=2 in adults); and who can be followed carefully long term for reactivation; and if there is evidence of HBeAg loss and seroconversion to anti-HBe (in persons initially HBeAg-positive) and after completion of at least one additional year of treatment; and in association with persistently normal ALT levels and persistently undetectable HBV DNA levels (where testing is available).  2.Where HBV DNA testing is not available: persons who have evidence of persistent HBsAg loss and after completion of at least one additional year of treatment, regardless of prior HBeAg status. |

Abbreviations: AASLD, the American Association for the Study of Liver Diseases; ALT, alanine aminotransferase; Anti-HBs, antibodies to HBsAg; APASL, the Asian Pacific Association for the Study of the Liver; CHB, chronic hepatitis B; EASL, European Association for the Study of the Liver; HBeAg, hepatitis B e antigen; HBsAg, hepatitis B surface antigen; HBV, hepatitis B virus C; NAs, nucleos(t)ide analogues; ULN, upper limit of normal.
